# Reduced circulating endothelial progenitor cells in reversible cerebral vasoconstriction syndrome

**DOI:** 10.1186/1129-2377-15-82

**Published:** 2014-12-02

**Authors:** Shih-Pin Chen, Yen-Feng Wang, Po-Hsun Huang, Chin-Wen Chi, Jong-Ling Fuh, Shuu-Jiun Wang

**Affiliations:** 1 Faculty of Medicine, School of Medicine, National Yang-Ming University, Taipei, Taiwan; 2 Department of Neurology, Neurological Institute, Taipei Veterans General Hospital, Taipei, Taiwan; 3 Brain Research Center, National Yang-Ming University, Taipei, Taiwan; 4 Institute of Brain Science, National Yang-Ming University, Taipei, Taiwan; 5 Department of Internal Medicine, Division of Cardiology, Taipei Veterans General Hospital, Taipei, Taiwan; 6 Department and Institute of Pharmacology, School of Medicine, National Yang-Ming University, Taipei, Taiwan; 7 Department of Medical Research & Education, Taipei Veterans General Hospital, Taipei, Taiwan

**Keywords:** Reversible cerebral vasoconstriction syndrome, Thunderclap headaches, Endothelial progenitor cells, Endothelial dysfunction, Cerebral arteries

## Abstract

**Background:**

The pathophysiology of reversible cerebral vasoconstriction syndrome (RCVS) remains elusive. Endothelial dysfunction might play a role, but direct evidence is lacking. This study aimed to explore whether patients with RCVS have a reduced level of circulating circulating endothelial progenitor cells (EPCs) to repair the dysfunctional endothelial vasomotor control.

**Methods:**

We prospectively recruited 24 patients with RCVS within one month of disease onset and 24 healthy age- and sex-matched controls. Flow cytometry was used to quantify the numbers of circulating EPCs, defined as KDR^+^CD133^+^, CD34^+^CD133^+^, and CD34^+^KDR^+^ double-positive mononuclear cells. The Lindegaard index, an index of vasoconstriction, was calculated by measuring the mean flow velocity of middle cerebral arteries and distal extracranial internal carotid arteries via color-coded sonography on the same day as blood drawing. A Lindegaard index of 2 was chosen as the cutoff value for significant vasoconstriction of middle cerebral arteries based on our previous study.

**Results:**

Patients with RCVS had a reduced number of CD34^+^KDR^+^ cells (0.009 ± 0.006% vs. 0.014 ± 0.010%, p = 0.031) but not KDR^+^CD133^+^ cells or CD34^+^CD133^+^ EPCs, in comparison with controls. The number of CD34^+^KDR^+^ cells was inversely correlated with the Lindegaard index (rs = -0.418, p = 0.047). Of note, compared to controls, patients with a Lindegaard index > 2 (n = 13) had a reduced number of CD34^+^KDR^+^ cells (0.007 ± 0.005% vs. 0.014 ± 0.010%, p = 0.010), but those with a Lindegaard index ≤ 2 did not.

**Conclusions:**

Patients with RCVS had reduced circulating CD34^+^KDR^+^ EPCs, which were correlated with the severity of vasoconstriction. Endothelial dysfunction might contribute to the pathogenesis of RCVS.

## Background

Reversible cerebral vasoconstriction syndrome (RCVS) is clinical-radiological syndrome characterized by recurrent thunderclap headaches and reversible segmental vasoconstrictions of cerebral arteries [1,2]. Large case series have demonstrated that RCVS is not uncommon [3-5], but rather an under-recognized clinical emergency [6]. Patients with RCVS exhibit substantial risks of devastating complications such as posterior reversible encephalopathy syndrome (PRES), ischemic stroke, and intracranial hemorrhages (including cortical subarachnoid, intracerebral, and even subdural hemorrhage) [3-5,7-9].

RCVS can be either idiopathic or secondary. For some secondary RCVS cases, the use of cocaine or marijuana is the inciting factor responsible for the pathogenesis. However, the pathogenesis of idiopathic RCVS is enigmatic. Sympathetic overactivity [10], oxidative stress [11] and genetic predisposition [12] might play certain roles. We suspect that endothelial dysfunction in RCVS is also highly plausible because both sympathetic overactivity and oxidative stress are detrimental to the endothelium [13-15]. Recent studies showed that bone marrow-derived endothelial progenitor cells (EPCs) are the main source that contributes to the regeneration and maintenance of the endothelium [16,17]. The number of circulating EPCs has been reported to be a surrogate biologic marker of vascular function and to correlate inversely with the endothelial repair capacity and cardiovascular risks [17-19]. We hypothesized that patients with RCVS might have decreased circulating EPCs during the course of vasoconstriction.

## Methods

### Ethics

The study protocol was approved by the Institutional Review Board of Taipei Veterans General Hospital. All participants provided written informed consent before entering the study. All clinical investigations were conducted according to the principles expressed in the Declaration of Helsinki. The corresponding authors had full access to all of the data in the study and had final responsibility for the decision to submit the results for publication.

### Participants and clinical settings

Patients with RCVS were recruited from the Neurology Department of Taipei Veterans General Hospital, a 2,909-bed national medical center located in Taipei City from 2011 to 2013. Age- and sex-matched volunteer participants who had neither headache history nor severe medical illness were recruited as normal controls. The inclusion criteria for the participants (both patients and controls) were the following: (1) age between 20 and 65 years, and (2) subjects could fully understand the purpose of the research and voluntarily join the study. Because many intrinsic or exogenous factors could influence the number of circulating EPCs, we applied very strict exclusion criteria and selectively enrolled matched controls to eliminate the influence of these confounders. Subjects were excluded if they met the following criteria: (1) had a smoking history, (2) had a major systemic illness such as uncontrolled hypertension (systolic blood pressure > 160 mmHg, diastolic blood pressure > 100 mmHg) at baseline, diabetes mellitus, cardiovascular or cerebrovascular disease, chronic hepatic or renal disease, or malignancies, (3) used illicit drugs; or (4) women who were pregnant or within 3 months postpartum. Subjects with a history of migraine and grade 1 hypertension (systolic blood pressure 140–159 mmHg, diastolic blood pressure 90–99 mmHg) were permitted to enroll because some patients with RCVS may have migraine and hypertension.

The diagnosis of RCVS was based on the following criteria: (1) at least two acute-onset severe headaches (thunderclap headaches), with or without focal neurological deficits; (2) vasoconstrictions demonstrated on magnetic resonance angiography (MRA); (3) reversibility of vasoconstrictions, as demonstrated by at least one follow-up MRA within 3 months; and (4) aneurysmal subarachnoid hemorrhage or other intracranial disorders were ruled out by appropriate investigations, but cortical subarachnoid hemorrhage in RCVS was allowed. The diagnostic criteria were adapted from the criteria of "benign (or reversible) angiopathy of the central nervous system" proposed by the International Classification of Headache Disorders, second edition (ICHD-2) (Code 6.7.3) [20] with the exception of the duration criterion D as well as the essential diagnostic elements of RCVS proposed by Calabrese *et al*. [1]. In this study, only patients with idiopathic RCVS within one month of headache onset were eligible to minimize potential confounders.

### Clinical evaluations

Basic demographic information and anthropometric measurements including height, body weight, and body mass index (BMI) [21] were collected. All enrolled subjects received comprehensive clinical and neurological examinations, and provided detailed medical, headache, and drug histories upon entering the study. Lipid profiles were obtained from general serological surveys. Diagnostic investigations including the first MRA, transcranial color-coded sonography and/or spinal taps were performed within the first two days after the patient was seen. Control subjects did not receive neuroimaging studies. The protocols have been reported previously [3,7,8]. In brief, brain MRI, MRA and MR venography (MRV) studies were performed with a 3-Tesla MR scanner. Intracranial lesions were carefully evaluated with adequate MR sequences, for example, subarachnoid hemorrhage with susceptibility weighted imaging (SWI) and intracranial aneurysms with three-dimensional time-of flight (TOF) MRA. Sequential MRAs were performed in all subjects with RCVS until the normalization of vasoconstrictions. The mean flow velocity of the major cerebral arteries was detected by transcranial color-coded sonography and recorded; the protocols were detailed elsewhere [7]. The following data were analyzed: mean flow velocity of middle cerebral artery (MCA) (V_MCA_) and the Lindegaard index (LI), which is calculated by dividing the V_MCA_ by the mean flow velocity of the ipsilateral distal extracranial internal carotid artery. The LI was selected to evaluate the vasoconstrictions to avoid possible misreading of hyperemia as vasospasm. For computational purposes, the average V_MCA_ and LI derived from the bilateral MCAs in each patient were calculated. We analyzed only V_MCA_ and LI because MCA is the most widely studied and validated vessel for vasoconstrictions using transcranial color-coded sonography (TCCS) and there have been no validated sonographic criteria of vasoconstrictions for the other cerebral vessels with adequate sensitivity [7]. In addition, our previous studies showed a good correlation of V_MCA_ and LI with the severity of vasoconstrictions detected by MRA.

Because the severity of vasoconstriction of the MCA could vary between patients at the time of blood sampling, we further stratified the patients according to their LI values. Based on our previous study [7], an LI value of 2, the upper limit of our normal controls, was chosen to stratify the patients into two groups: those having either significant or insignificant vasoconstrictions of the MCA. An LI value of 3 was also used to evaluate whether patients at risk of ischemic complications [7] could have lower EPCs. Of note, the cut-off values of LI used here were based on our previous study in RCVS patients using TCCS [7] and should not be translated to the same severity of vasopasm in SAH based on studies using transcranial Doppler (TCD) [22]. In patients with SAH, the LI is disproportionately amplified because of low extracranial ICA flow velocity due to cerebral hypoperfusion caused by increased intracranial pressure (IICP) [22,23], while patients with RCVS usually have normal ICP [1-3]. Patients with RCVS who fulfilled the mild vasospasm criteria for SAH already had high risks of developing PRES (75%) or ischemic stroke (50%) [7].

### Flow cytometric analysis of circulating EPCs

The flow cytometric analysis of circulating EPCs was performed based on our previously established methods [24]. Peripheral blood was collected from the antecubital vein on the same day of transcranial color-coded sonography, before administration of any therapeutics. The samples were processed immediately after blood drawing. A volume of 100 μL blood was incubated for 15 minutes in the dark with monoclonal antibodies against human kinase insert domain receptor (KDR; R&D, Minneapolis, MN, USA) followed by phycoerythrin (PE)-conjugated secondary antibody, with the fluorescein isothiocyanate (FITC)-labeled monoclonal antibodies against human CD45 (Becton Dickinson, Franklin Lakes, NJ, USA), with the PE-conjugated monoclonal antibody against human CD133 (Miltenyi Biotec, Germany), and with FITC-conjugated or PE-conjugated monoclonal antibodies against human CD34 (Serotec, Raleigh, NC, USA). Isotype-identical antibodies were used as controls (Becton Dickinson, Franklin Lakes, NJ, USA). After incubation, cells were lysed, washed with phosphate-buffered saline (PBS), and fixed in 2% paraformaldehyde before analysis. Each analysis included 100,000 events. Flow cytometry was used to quantify the numbers of circulating EPCs defined as KDR^+^CD133^+^, CD34^+^CD133^+^, and CD34^+^KDR^+^ double-positive mononuclear cells (Additional file 1: Figure S1). To assess the reproducibility of the EPC measurements, circulating EPCs were measured from two separate blood samples in 20 subjects (10 controls and 10 patients each). Mature circulating endothelial cells, which are generally recognized as cells expressing endothelial markers (CD 144, CD 146, vWF, VEGFR-2) in the absence of hematopoietic (CD 14, CD 45) and progenitor (CD 34, CD 133) markers (despite the lack of a clear concensus on their phenotypic definitions) [25,26], were excluded with this method.

### Statistics

Descriptive statistics are presented as the mean ± standard deviation or as the number (percentage). For normally distributed variables, continuous ones were compared by the Student’s t-test, and categorical ones were compared by the Chi-square or Fisher exact tests. For variables that don’t have a normal distribution, Kruskal-Wallis test and Mann–Whitney U test were used as appropriate. For correlations between EPC counts with other variables, Spearman correlation coefficient r_s_ was applied because EPC counts were not normally distributed (by Shapiro-Wilk test). All calculated *p*-values were two-tailed. Statistical significance was defined as *p* < 0.05. Because there was collinearity between the number of KDR^+^CD133^+^, CD34^+^CD133^+^, and CD34^+^KDR^+^ cells (the correlation coefficients were 0.666 for CD34^+^CD133^+^ and CD34^+^KDR^+^ cells (p < 0.001), 0.735 for KDR^+^CD133^+^ and CD34^+^CD133^+^ cells (p < 0.001), and 0.570 for KDR^+^CD133^+^ and CD34^+^KDR^+^ cells (p < 0.001)), corrections for multiple comparisons were not applied in this study. All analyses were performed with the IBM SPSS Statistics software package, version 18.0.

## Results

### Participants and their characteristics

During the two-year study period, we prospectively recruited 31 patients with RCVS. After excluding one patient with a previous history of intracerebral hemorrhage, one patient with previous cerebral infarction, two patients who used psueodephedrine, one patient who had a history of marijuana use, one patient who was postpartum, and one heavy smoker, 24 patients were eligible for study. Another 24 age- and sex-matched controls were recruited during the study period. There was no difference in the demographics, BMIs, medical illnesses, and serum cholesterol levels between the two groups (Table 1). Most of the patients (91.7%) had identifiable triggers for their thunderclap headaches, with defecation (58.3%) and bathing (29.2%) being the most common. The cerebrospinal fluid was studied in 12 (50%) patients, and their results were all normal.

**Table 1:** Basic demongraphics, body mass index, and medical illness of participants

	**Controls(n = 24)**	**RCVS(n = 24)**	**P value**
Age, y (mean ± SD)	45.0 ± 11.9	48.8 ± 9.5	0.229
Gender (M/F)	4/20	4/20	1.000
Menopause, n (%)	6 (12%)	7 (14%)	0.906
Body mass index (kg/m^2^)	25.3 ± 1.7	24.0 ± 3.0	0.525
Hypertension, n (%)	0 (0%)	1 (4.2%)	0.312
Diabetes, n (%)	0 (%)	0 (0%)	1.000
Coronary artery disease, n (%)	0 (%)	0 (0%)	1.000
Migraine, n (%)	2 (8.3%)	4 (16.7%)	0.663
Cholesterol (mg/dL)	193.6 ± 35.3	183.7 ± 28.7	0.370

### Neuroimaging findings

None of the patients had posterior reversible encephalopathy syndromes, ischemic stroke, intracerebral hemorrhage or intracranial arterial dissection on the initial or follow-up MR studies, but one patient had cortical subarachnoid hemorrhage confined to right anterior high frontal cortex without identifiable aneurysm. None of the patients had aneurysmal subarachnoid hemorrhage in consecutive MR studies.

### EPC measurements

The blood samples were taken at a mean of 5.4 ± 3.7 days (range 2–13) after headache onset. Of the 20 subjects who had two separate blood samples, there was a strong correlation between the two measurements suggesting high reproducibility (CD34^+^CD133^+^: rs = 0.973, *p* < 0.001; CD34^+^KDR^+^: rs = 0.972, *p* < 0.001; and KDR^+^CD133^+^: rs = 0.976, *p* < 0.001). There were no correlations between the EPC counts and age, gender, BMI, menopausal status, migraine, hypertension, or serum cholesterol level.

In comparison to the controls, patients with RCVS had reduced numbers of CD34^+^KDR^+^ cells (0.009 ± 0.006% vs. 0.014 ± 0.010%, *p* = 0.031), but they had no reduction in KDR^+^CD133^+^ cells (0.031 ± 0.015% vs. 0.039 ± 0.020%, *p* = 0.176) or CD34^+^CD133^+^ EPCs (0.045 ± 0.028% vs. 0.060 ± 0.041%, *p* = 0.140). The EPC counts in patients with comorbid migraine (n = 4) did not differ from that in patients without comorbid migraine (n = 20) (CD34^+^KDR^+^ cells (0.007 ± 0.006% vs. 0.009 ± 0.006%, *p* = 0.751), KDR^+^CD133^+^ cells (0.027 ± 0.010% vs. 0.032 ± 0.015%, *p* = 0.642), CD34^+^CD133^+^ EPCs (0.042 ± 0.007% vs. 0.045 ± 0.030%, *p* = 0.781)).

**Figure 1:**
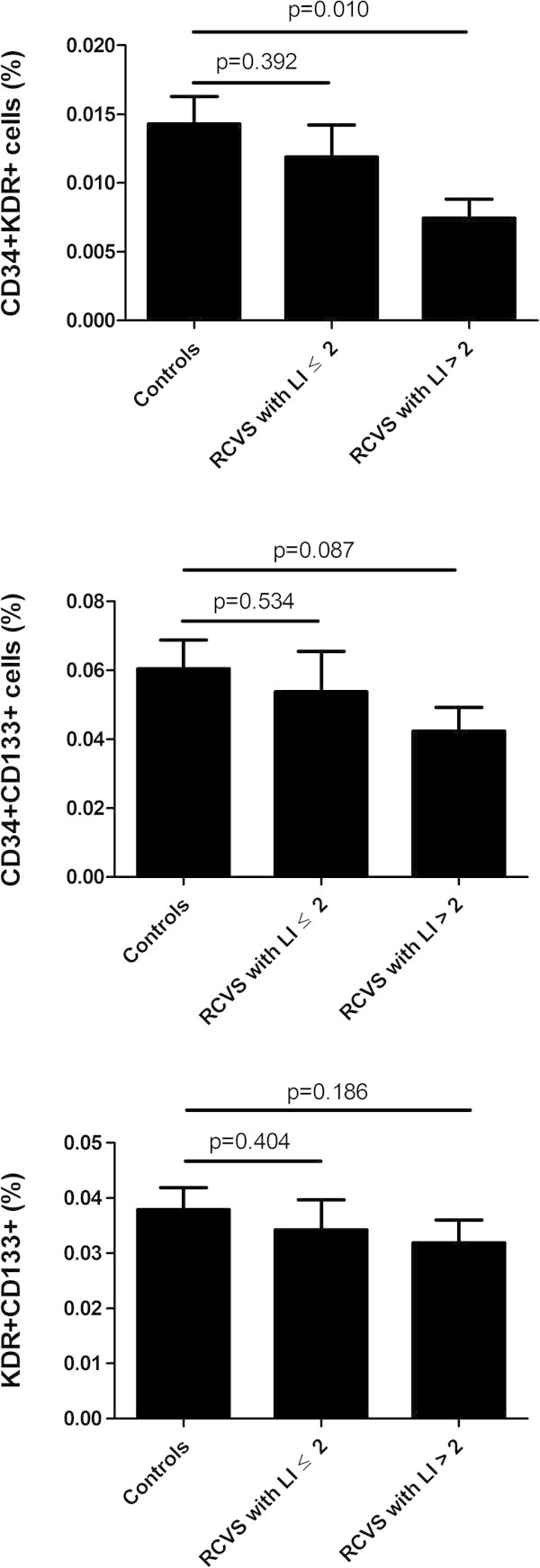
**Comparison of the numbers of endothelial progenitor cells between patients with reversible cerebral vasoconstriction syndrome and controls.** (column: mean, error bars: standard errors). RCVS: reversible cerebral vasoconstriction syndrome; LI: Lindegaard index.

In comparison with the controls, patients with an LI > 2 (n = 13) had fewer CD34^+^KDR^+^ cells (0.007 ± 0.005% vs. 0.014 ± 0.010%, *p* = 0.010), but there was no difference in the number of KDR^+^CD133^+^ cells (0.032 ± 0.014% vs. 0.039 ± 0.020%, *p* = 0.186) or CD34^+^CD133^+^ EPCs (0.042 ± 0.023% vs. 0.060 ± 0.041%, *p* = 0.087).The EPC counts of patients with LI ≤ 2 did not differ from those of the controls (CD34^+^KDR^+^ cells: 0.012 ± 0.006% vs. 0.014 ± 0.010%, *p* = 0.392; KDR^+^CD133^+^ cells: 0.036 ± 0.016% vs. 0.039 ± 0.020%, *p* = 0.404; CD34^+^CD133^+^ cells: 0.052 ± 0.032% vs. 0.060 ± 0.041%, *p* = 0.534.) (Figure 1)

In patients with LI > 3 (n = 3), the trend was similar: Their CD34^+^KDR^+^ cell counts were lower than the controls (0.006 ± 0.002% vs. 0.014 ± 0.010%, *p* = 0.015), but there was no difference in the numbers of KDR^+^CD133^+^ cells (0.023 ± 0.008% vs. 0.039 ± 0.020%, *p* = 0.070) or CD34^+^CD133^+^ EPCs (0.032 ± 0.012% vs. 0.060 ± 0.041%, *p* = 0.054).

### Correlations between EPCs and clinical variables

**Figure 2:**
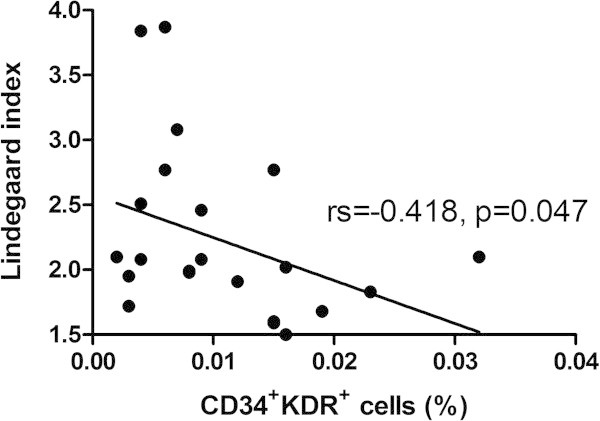
**Correlation between the numbers of CD34**^**+**^**KDR**^**+ **^**endothelial progenitor cells and Lindegaard index.**

The number of CD34^+^KDR^+^ cells was negatively correlated with the LI (rs = -0.418, *p* = 0.047) (Figure 2), whereas there was no significant correlation of LI with the number of KDR^+^CD133^+^ (rs = -0.265, *p* = 0.221) or CD34^+^CD133^+^ cells (rs = -0.207, *p* = 0.344). None of the other clinical variables such as triggers (including defecation, bathing, exertion, cough, rage, or sex) or the number and duration of thunderclap headaches before blood drawing had significant correlations with the EPCs counts (data not shown).

## Discussions

Our study demonstrated that patients with RCVS had reduced circulating CD34^+^KDR^+^ EPCs in comparison with controls, especially in those with more severe vasoconstrictions (LI > 2) and those at risk of ischemic complications (LI > 3). The number of CD34^+^KDR^+^ EPCs was negatively correlated with the severity of vasoconstrictions evaluated with the LI. In contrast, the KDR^+^CD133^+^ and CD34^+^CD133^+^ EPCs did not show any associations with RCVS.

Circulating EPCs are capable of patching sites of denuding injury, and they serve as a cellular reservoir for the replacement of dysfunctional endothelium [27,28]. However, it is unclear which antigenic profiles best identify progenitor cells with the potential for endothelial repair [29]. Cells expressing CD34 (an adhesion molecule expressed on hematopoietic stem cells) and KDR (a type 2 vascular endothelial growth factor receptor that indicates early endothelial differentiation) might be the main constituent of the circulating EPCs responsible for re-endothelialization [30]. The identification of fewer circulating CD34^+^KDR^+^ EPCs was consistent with our hypothesis that patients with RCVS had a reduced capacity for endothelial repair. The inverse correlation between the CD34^+^KDR^+^ EPC count and the severity of vasoconstriction further supports the hypothesis that the reduced endothelial repair capability might be associated with the overwhelmed vasomotor control in RCVS. The reason why the KDR^+^CD133^+^ and CD34^+^CD133^+^ EPCs were not correlated with severity of vasoconstrictions was unknown, but these findings might suggest that not all the EPC phenotypes were associated with vasomotor control.

It is unclear whether there is a causal relationship between reduced EPC counts and vasoconstrictions in RCVS. Whether patients with RCVS have already had reduced circulating EPCs before disease onset could never be proved since it’s almost impossible to have pre-morbid EPC measurement. We put forth the following hypotheses. First, it is possible that when an unknown insult ignites the vicious cycle of sympathetic overactivity [10] and oxidative stress [11], which damages the endothelium of the cerebral vasculature, EPCs will be mobilized from the bone marrow into the circulation to repair the endothelial damage. When patients have low baseline EPCs or a compromised capacity of EPC mobilization, they are likely to develop more severe vasoconstriction. In contrast, patients with sufficient EPCs might have less severe vasoconstrictions. Second, sympathetic overactivity and oxidative stress may accelerate the consumption or senescence of circulating EPCs [31,32], which could further hamper the numbers and function of EPCs. However, this was purely speculative because we did not perform functional assays on EPCs in this study. Previous studies have shown that headache disorders such as migraine or cluster headache could be modulated by immunological cells that migrate from peripheral blood circulation into central nervous system, via an altered expression of adhesion molecules and chemotactic cytokines. Although mechanistically different, this neuroimmunological interplay might serve as a model for us to envision the mechanism of EPCs in RCVS [33,34].

Our study has limitations. First, to eliminate the influence of potential confounders that might interfere EPC counts, we applied very strict exclusion criteria and selectively enrolled matched controls. Hence, it is not possible to conclude whether the study results are generalizable to patients who have multiple vascular risk factors or obvious secondary causes of RCVS, such as illicit drug users. In addition, the number of participants was limited in this study partially due to the exclusion and matching criteria. However, considering the prevalence of RCVS, the possible interassay variability during the prolonged study duration (which could be due to machines, operators, and reagents, etc.), and the significant study results, we believe that the current case numbers are appropriate for our research purposes. Finally, as mentioned above, we did not perform functional assays on EPCs. Further mechanistic approaches are required.

## Conclusions

Patients with RCVS had reduced circulating CD34^+^KDR^+^ EPCs, which were inversely correlated with the severity of vasoconstrictions. A reduced capacity of circulating EPCs to repair dysfunctional endothelial vasomotor control might contribute to the pathogenesis of vasoconstrictions in patients with RCVS. The potential of therapies targeting the restoration of endothelial function deserves exploration.

## Abbreviations

BMI: Body mass index; EPC: Endothelial progenitor cell; ICHD-2: International Classification of Headache Disorders, second edition; IICP: Increased intracranial pressure; LI: Lindegaard index; MCA: Middle cerebral artery; MRA: Magnetic resonance angiography; MRV: Magnetic resonance venography; PRES: Posterior reversible encephalopathy syndrome; RCVS: Reversible cerebral vasoconstriction syndrome; SAH: Subarachnoid hemorrhage; TCCS: Transcranial color-coded sonography; TCD: Transcranial Doppler; V_MCA_: Mean flow velocity of middle cerebral artery.

## Competing interests

The authors declare that they have no competing interests.

## Authors’ contributions

Dr. SPC - Study concept and design, patient recruitment, acquisition of data, analysis and interpretation, manuscript writing. Dr. JLF - Study concept and design, patient recruitment, study supervision, critical revision of the manuscript for important intellectual content. Dr. SJW - Study concept and design, patient recruitment, study supervision, critical revision of the manuscript for important intellectual content. Dr. YFW - patient recruitment, acquisition of data, analysis and interpretation. Dr. PHH - technical support, critical revision of the manuscript for important intellectual content. Dr. CWC - technical support, critical revision of the manuscript for important intellectual content. All authors read and approved the final manuscript.

## Study funding

This study was supported by grants from the National Science Council of Taiwan (99-2314-B-075-036-MY3, 100-2314-B-010-019-MY2, 100-2314-B-010-018-MY3), Taipei-Veterans General Hospital (V100B-007, VGHUST101-G7-1-1, V101C-106, V101E7-003), NSC support for Center for Dynamical Biomarkers and Translational Medicine, National Central University, Taiwan (NSC 100-2911-I-008-001), Brain Research Center, National Yang-Ming University and a grant from Ministry of Education, Aim for the Top University Plan. No additional external funding received for this study. The funders had no role in study design, data collection and analysis, decision to publish, or preparation of the manuscript.

## Supplementary Material

Additional file 1: Figure S1 Representative flow cytometric analysis for quantifying the number of circulating endothelial progenitor cells (EPCs). Mononuclear cells were gated by forward/sideward scatter (FSC/SSC), and the numbers of circulating EPCs were defined as CD34^+^KDR^+^, CD34^+^CD133^+^, and KDR^+^CD133^+^ cells respectively. (A) Patients with reversible cerebral vasoconstriction syndrome, (B) Controls. Click here for file
