# Supplementary material for: Reduced circulating endothelial progenitor cells in reversible cerebral vasoconstriction syndrome
Source: J Headache Pain. 2014 Dec 2;15(1):82. doi: 10.1186/1129-2377-15-82 (PMC4266547; doi:10.1186/1129-2377-15-82)

**Figure S1. Representative flow cytometric analysis for quantifying the number of circulating endothelial progenitor cells (EPCs).**

Mononuclear cells were gated by forward/sideward scatter (FSC/SSC), and the numbers of circulating EPCs were defined as CD34^+^KDR^+^, CD34^+^CD133^+^, and KDR^+^CD133^+^ cells respectively. (A) Patients with reversible cerebral vasoconstriction syndrome, (B) Controls.


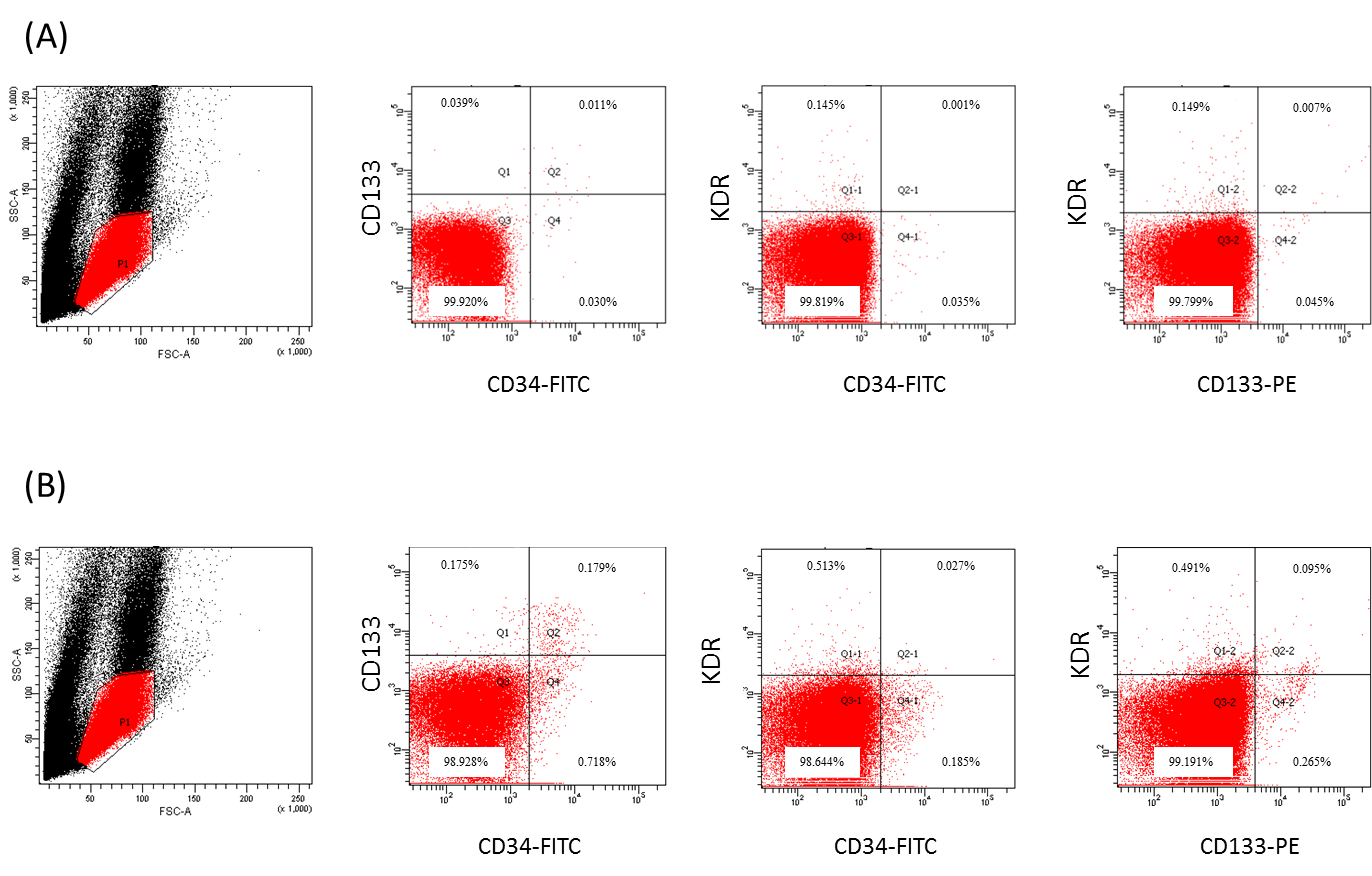

Supplement: Additional file 1: Figure S1 — Representative flow cytometric analysis for quantifying the number of circulating endothelial progenitor cells (EPCs). Mononuclear cells were gated by forward/sideward scatter (FSC/SSC), and the numbers of circulating EPCs were defined as CD34+KDR+, CD34+CD133+, and KDR+CD133+ cells respectively. (A) Patients with reversible cerebral vasoconstriction syndrome, (B) Controls. [file 1129-2377-15-82-S1.docx]
